# *N*-(3,4-Dimethyl­phen­yl)maleamic acid

**DOI:** 10.1107/S1600536809043682

**Published:** 2009-10-28

**Authors:** B. Thimme Gowda, Miroslav Tokarčík, Jozef Kožíšek, K. Shakuntala, Hartmut Fuess

**Affiliations:** a Department of Chemistry, Mangalore University, Mangalagangotri 574 199, Mangalore, India; b Faculty of Chemical and Food Technology, Slovak Technical University, Radlinského 9, SK-812 37 Bratislava, Slovak Republic; c Institute of Materials Science, Darmstadt University of Technology, Petersenstrasse 23, D-64287 Darmstadt, Germany

## Abstract

The title compound, C_12_H_13_NO_3_, crystallizes with four independent mol­ecules in the asymmetric unit. The N—H bond and the C=O bond in the amide segment are *anti* to each other. The C=C double bond is *cis* configured and an intra­molecular O—H⋯O hydrogen bond is formed in each molecule. The mean planes through the aromatic ring and the amide group –NHCO– are inclined at angles of 17.4 (3), 20.8 (2), 16.2 (2) and 11.2 (3)° in the four mol­ecules. In the crystal, inter­molecular N—H⋯O hydrogen bonds link the mol­ecules into ribbons along the *b* axis.

## Related literature

For our study on the effect of ring and side-chain substitutions on the crystal structures of biologically important amides, see: Gowda, Foro, Saraswathi & Fuess (2009 ▶); Gowda, Foro, Saraswathi, Terao & Fuess (2009 ▶); Gowda, Tokarčík *et al.* (2009 ▶); Prasad *et al.* (2002 ▶). For modes of inter­linking carboxylic acids by hydrogen bonds, see: Leiserowitz (1976 ▶). For a related structure, see: Lo & Ng (2009 ▶). 
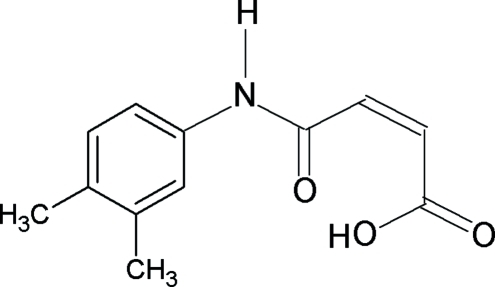


## Experimental

### 

#### Crystal data

C_12_H_13_NO_3_
*M* *_r_* = 219.23
Monoclinic, 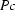

*a* = 11.9003 (2) Å
*b* = 12.9991 (2) Å
*c* = 15.2641 (3) Å
β = 110.207 (2)°
*V* = 2215.92 (7) Å^3^
*Z* = 8
Mo *K*α radiation
μ = 0.10 mm^−1^
*T* = 295 K
0.48 × 0.32 × 0.31 mm

#### Data collection

Oxford Diffraction Xcalibur Ruby Gemini diffractometer
Absorption correction: multi-scan (*CrysAlisPro*; Oxford Diffraction, 2009 ▶) *T*_min_ = 0.958, *T*_max_ = 0.965
67608 measured reflections
5279 independent reflections
4100 reflections with *I* > 2σ(*I*)
*R*_int_ = 0.028

#### Refinement

*R*[*F* ^2^ > 2σ(*F* ^2^)] = 0.044
*wR*(*F* ^2^) = 0.134
*S* = 1.03
5279 reflections
577 parameters
10 restraints
H-atom parameters constrained
Δρ_max_ = 0.29 e Å^−3^
Δρ_min_ = −0.16 e Å^−3^

### 

Data collection: *CrysAlis Pro* (Oxford Diffraction, 2009 ▶); cell refinement: *CrysAlis Pro*; data reduction: *CrysAlis Pro*; program(s) used to solve structure: *SHELXS97* (Sheldrick, 2008 ▶); program(s) used to refine structure: *SHELXL97* (Sheldrick, 2008 ▶); molecular graphics: *ORTEP-3* (Farrugia, 1997 ▶) and *DIAMOND* (Brandenburg, 2002 ▶); software used to prepare material for publication: *SHELXL97* (Sheldrick, 2008 ▶), *PLATON* (Spek, 2009 ▶) and *WinGX* (Farrugia, 1999 ▶).

## Supplementary Material

Crystal structure: contains datablocks I, global. DOI: 10.1107/S1600536809043682/bt5110sup1.cif

Structure factors: contains datablocks I. DOI: 10.1107/S1600536809043682/bt5110Isup2.hkl

Additional supplementary materials:  crystallographic information; 3D view; checkCIF report

## Figures and Tables

**Table 1:** Hydrogen-bond geometry (Å, °)

*D*—H⋯*A*	*D*—H	H⋯*A*	*D*⋯*A*	*D*—H⋯*A*
N1—H1⋯O63^i^	0.86	2.00	2.851 (3)	168
N2—H2*N*⋯O43	0.86	2.03	2.865 (3)	163
N3—H3*N*⋯O23^i^	0.86	2.07	2.916 (3)	167
N4—H4*N*⋯O3	0.86	2.08	2.930 (3)	169
O2—H2*A*⋯O1	0.88	1.62	2.481 (3)	165
O22—H22*A*⋯O21	0.88	1.59	2.471 (3)	176
O42—H42*A*⋯O41	0.88	1.61	2.487 (3)	175
O62—H62*A*⋯O61	0.88	1.6	2.480 (3)	176

Symmetry code: (i) 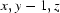.

**Fig. 1.**
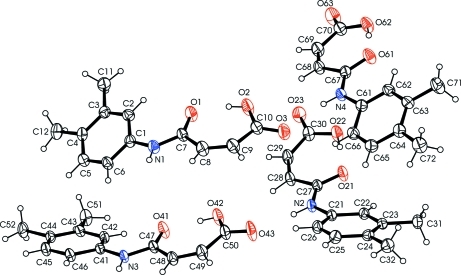
Molecular structure of the title compound showing the atom labelling scheme. Displacement ellipsoids are drawn at the 30% probability level. H atoms are represented as small spheres of arbitrary radii.

**Fig. 2.**
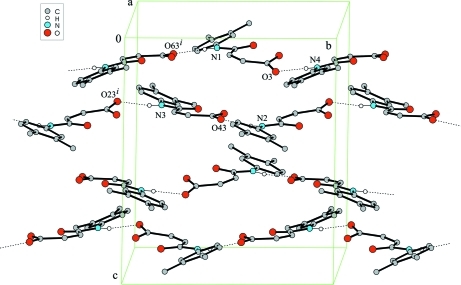
Part of crystal structure of the title compound with N—H···O hydrogen bonds represented by dashed lines. Symmetry code (i): x,y - 1,z. H atoms not involved in intermolecular hydrogen bonding have been omitted.

## supplementary crystallographic information

### Comment

As a part of studying the effect of ring and side chain substitutions on the crystal structures of biologically important amides (Gowda,  Foro,   Saraswathi  & Fuess, 2009; Gowda,  Foro, Saraswathi, Terao  & Fuess, 2009; Gowda, Tokarčík *et al.*, 2009; Prasad *et al.*, 2002), the crystal structure of *N*-(3,4-dimethylphenyl)-maleamic acid (I) has been determined. The asymmetric unit of the cell contains four independent molecules (Fig. 1). The conformations of the N—H and C=O bonds in the amide segment of the structure are *anti* to each other and those of the amide O atom and the carbonyl O atom of the acid segment are also *anti* to each other. But the amide O atom is *anti* to the H atom attached to the adjacent C atom, while the carboxyl O atom is *syn* to the H atom attached to its adjacent C atom (Fig.1). In the structure of (I), the rare *anti* conformation of the C=O and O—H bonds of the acid group has been observed, similar to that obsrved in *N*-(2,6-dimethylphenyl)maleamic acid (Gowda, Tokarčík *et al.*, 2009) and *N*-phenylmaleamic acid (Lo & Ng, 2009), but contrary to the more general *syn* conformation observed for C=O and O—H bonds of the acid group in *N*- (2,6-dimethylphenyl)succinamic acid (Gowda *et al.*, 2009*b*). The various modes of interlinking carboxylic acids by hydrogen bonds is described elsewhere (Leiserowitz, 1976).

In the maleamic moiety the C8—C9, C28—C29, C48—C49 and C68—C69 bond lengths of 1.322 (5), 1.343 (4), 1.320 (4) and 1.321 (4) Å clearly indicate the double bond character. Each maleamic moiety features one intramolecular hydrogen O–H···O bond (Table 1). The mean planes through the phenyl ring and the amido group –NHCO– are inclined at the angles of 17.4 (3), 20.8 (2), 16.2 (2) and 11.2 (3)° in the first, second, third and fourth molecules, respectively. In the crystal structure, the intermolecular N–H···O hydrogen bonds link the molecules into ribbons parallel to the *ab*-plane of the cell (Fig. 2).

### Experimental

To a solution of maleic anhydride (0.025 mol) in toluene (25 ml) was added dropwise a solution of 3,4-dimethylaniline (0.025 mol) also in toluene (20 ml) with constant stirring. The resulting mixture was warmed with stirring for over 30 min and set aside for   additional 30 min at room temperature for the completion of reaction. The mixture was then treated with dilute hydrochloric acid to remove the unreacted 3,4-dimethylaniline. The resultant solid *N*-(3,4-dimethylphenyl)maleamic acid was filtered under suction and washed thoroughly with water to remove the unreacted maleic anhydride and maleic acid. It was recrystallized to constant melting point from ethanol. The purity of the compound was checked by elemental analysis and characterized by its infrared spectra. The single crystals used in X-ray diffraction studies were grown in an ethanol solution by slow evaporation at room temperature.

### Refinement

All H atoms bonded to C and N atoms were positioned with idealized geometry (C—H = 0.93 or 0.96 Å, N—H = 0.86 Å) and refined using a riding model. H atoms of the carboxyl groups were located in a difference map and finally refined with O—H distance fixed at 0.88 Å. The *U*_iso_(H) values were set at 1.2*U*_eq_(C_aromatic_, N, O) and 1.5*U*_eq_(C_methyl_). Two methyl groups (C12 and C72) exhibit orientational disorder in hydrogen atoms positions. In both groups two sets of H atoms were refined with equal occupancies of 0.50. The *U* values of the fragment C62, C63, C64, C65, C66 and C72 and of the atom pairs C42—C43 and N2—C21 were subject to a restraint (DELU instruction), *i.e.* the components of the displacement parameters in the direction of the bond were restrained to be equal within an effective standard deviation 0.005. Because of low anomalous scattering power the absolute structure cannot be determined reliably and therefore 5143 Friedel pairs were merged.

### Crystal data

**Table:**

C_12_H_13_NO_3_	*F*(000) = 928
*M**_r_* = 219.23	*D*_x_ = 1.314 Mg m^−^^3^
Monoclinic, *P**c*	Mo *K*α radiation, λ = 0.71073 Å
Hall symbol: P -2yc	Cell parameters from 31828 reflections
*a* = 11.9003 (2) Å	θ = 1.9–27.8°
*b* = 12.9991 (2) Å	µ = 0.10 mm^−^^1^
*c* = 15.2641 (3) Å	*T* = 295 K
β = 110.207 (2)°	Truncated square pyramid, colourless
*V* = 2215.92 (7) Å^3^	0.48 × 0.32 × 0.31 mm
*Z* = 8	

### Data collection

**Table:**

Oxford Diffraction Xcalibur Ruby Gemini diffractometer	5279 independent reflections
graphite	4100 reflections with *I* > 2σ(*I*)
Detector resolution: 10.434 pixels mm^-1^	*R*_int_ = 0.028
ω scans	θ_max_ = 27.9°, θ_min_ = 2.1°
Absorption correction: multi-scan (*CrysAlis PRO*; Oxford Diffraction, 2009)	*h* = −15→15
*T*_min_ = 0.958, *T*_max_ = 0.965	*k* = −17→17
67608 measured reflections	*l* = −20→20

### Refinement

**Table:**

Refinement on *F*^2^	Primary atom site location: structure-invariant direct methods
Least-squares matrix: full	Secondary atom site location: difference Fourier map
*R*[*F*^2^ > 2σ(*F*^2^)] = 0.044	Hydrogen site location: inferred from neighbouring sites
*wR*(*F*^2^) = 0.134	H-atom parameters constrained
*S* = 1.03	*w* = 1/[σ^2^(*F*_o_^2^) + (0.0958*P*)^2^ + 0.0272*P*] where *P* = (*F*_o_^2^ + 2*F*_c_^2^)/3
5279 reflections	(Δ/σ)_max_ = 0.001
577 parameters	Δρ_max_ = 0.29 e Å^−^^3^
10 restraints	Δρ_min_ = −0.16 e Å^−^^3^

### Special details

**Table:**

Geometry. All e.s.d.'s (except the e.s.d. in the dihedral angle between two l.s. planes) are estimated using the full covariance matrix. The cell e.s.d.'s are taken into account individually in the estimation of e.s.d.'s in distances, angles and torsion angles; correlations between e.s.d.'s in cell parameters are only used when they are defined by crystal symmetry. An approximate (isotropic) treatment of cell e.s.d.'s is used for estimating e.s.d.'s involving l.s. planes.
Refinement. Refinement of *F*^2^ against ALL reflections. The weighted *R*-factor *wR* and goodness of fit *S* are based on *F*^2^, conventional *R*-factors *R* are based on *F*, with *F* set to zero for negative *F*^2^. The threshold expression of *F*^2^ > σ(*F*^2^) is used only for calculating *R*-factors(gt) *etc*. and is not relevant to the choice of reflections for refinement. *R*-factors based on *F*^2^ are statistically about twice as large as those based on *F*, and *R*- factors based on ALL data will be even larger.

### Fractional atomic coordinates and isotropic or equivalent isotropic displacement parameters (Å^2^)

**Table:**

	*x*	*y*	*z*	*U*_iso_*/*U*_eq_	Occ. (<1)
C1	0.5468 (2)	0.4000 (2)	0.1080 (2)	0.0430 (6)	
C2	0.6117 (3)	0.4743 (2)	0.0804 (2)	0.0450 (6)	
H2	0.5758	0.5365	0.056	0.054*	
C3	0.7309 (3)	0.4559 (2)	0.0893 (2)	0.0462 (7)	
C4	0.7851 (3)	0.3631 (2)	0.1243 (2)	0.0474 (7)	
C5	0.7189 (3)	0.2888 (2)	0.1513 (2)	0.0493 (7)	
H5	0.7544	0.2264	0.1755	0.059*	
C6	0.6006 (3)	0.3075 (2)	0.1423 (2)	0.0484 (7)	
H6	0.5571	0.2569	0.1596	0.058*	
C7	0.3634 (3)	0.4981 (2)	0.0983 (2)	0.0468 (6)	
C8	0.2429 (3)	0.4810 (3)	0.1042 (3)	0.0564 (8)	
H8	0.2164	0.4132	0.0976	0.068*	
C9	0.1684 (3)	0.5495 (2)	0.1176 (3)	0.0585 (8)	
H9	0.0983	0.5213	0.1212	0.07*	
C10	0.1759 (3)	0.6647 (2)	0.1281 (2)	0.0524 (7)	
C11	0.7982 (3)	0.5385 (2)	0.0590 (3)	0.0589 (8)	
H11A	0.8646	0.5613	0.1118	0.088*	
H11B	0.7457	0.5955	0.0336	0.088*	
H11C	0.8272	0.5115	0.0123	0.088*	
C12	0.9124 (3)	0.3412 (3)	0.1342 (3)	0.0626 (9)	
H12A	0.9338	0.2734	0.1594	0.094*	0.5
H12B	0.9639	0.3909	0.1754	0.094*	0.5
H12C	0.9211	0.3453	0.074	0.094*	0.5
H12D	0.9455	0.3996	0.1131	0.094*	0.5
H12E	0.9153	0.2822	0.0972	0.094*	0.5
H12F	0.9581	0.3278	0.1985	0.094*	0.5
N1	0.4259 (2)	0.41119 (17)	0.10310 (19)	0.0495 (6)	
H1	0.3879	0.3549	0.1032	0.059*	
O1	0.40148 (19)	0.58444 (16)	0.08902 (19)	0.0609 (6)	
O2	0.2602 (2)	0.71471 (17)	0.1095 (2)	0.0745 (7)	
H2A	0.3048	0.671	0.092	0.089*	
O3	0.1018 (2)	0.70943 (18)	0.1511 (2)	0.0708 (7)	
C21	−0.0264 (2)	0.63766 (18)	0.36357 (17)	0.0369 (5)	
C22	−0.0921 (3)	0.7141 (2)	0.38831 (19)	0.0388 (6)	
H22	−0.0562	0.7768	0.4112	0.047*	
C23	−0.2114 (2)	0.69692 (19)	0.37893 (18)	0.0382 (6)	
C24	−0.2663 (2)	0.6027 (2)	0.34333 (18)	0.0405 (6)	
C25	−0.1979 (3)	0.5278 (2)	0.3203 (2)	0.0480 (7)	
H25	−0.2328	0.4647	0.2978	0.058*	
C26	−0.0802 (3)	0.5444 (2)	0.3298 (2)	0.0471 (7)	
H26	−0.0366	0.4931	0.3135	0.056*	
C27	0.1559 (3)	0.7351 (2)	0.3711 (2)	0.0429 (6)	
C28	0.2785 (3)	0.7178 (2)	0.3698 (2)	0.0505 (7)	
H28	0.3061	0.6503	0.3785	0.061*	
C29	0.3543 (3)	0.7882 (2)	0.3577 (3)	0.0552 (8)	
H29	0.4274	0.7609	0.3597	0.066*	
C30	0.3450 (3)	0.8997 (2)	0.3418 (2)	0.0511 (7)	
C31	−0.2811 (3)	0.7797 (2)	0.4058 (2)	0.0525 (7)	
H31A	−0.2289	0.8363	0.4332	0.079*	
H31B	−0.3142	0.7529	0.4503	0.079*	
H31C	−0.3447	0.8029	0.3513	0.079*	
C32	−0.3959 (3)	0.5826 (3)	0.3293 (2)	0.0548 (7)	
H32A	−0.415	0.5124	0.3107	0.082*	
H32B	−0.4455	0.6275	0.2816	0.082*	
H32C	−0.41	0.5952	0.3866	0.082*	
N2	0.09531 (19)	0.64905 (16)	0.37116 (16)	0.0417 (5)	
H2N	0.1352	0.5928	0.3765	0.05*	
O21	0.11403 (19)	0.82277 (15)	0.37276 (19)	0.0594 (6)	
O22	0.2552 (2)	0.95070 (16)	0.3502 (2)	0.0701 (8)	
H22A	0.2037	0.9074	0.3597	0.084*	
O23	0.4227 (2)	0.94389 (17)	0.3212 (2)	0.0689 (7)	
C41	0.6529 (2)	0.13584 (19)	0.35178 (18)	0.0367 (5)	
C42	0.7424 (3)	0.2090 (2)	0.3831 (2)	0.0413 (6)	
H42	0.7261	0.2714	0.406	0.05*	
C43	0.8552 (2)	0.1909 (2)	0.38079 (19)	0.0400 (5)	
C44	0.8820 (2)	0.0970 (2)	0.3492 (2)	0.0408 (6)	
C45	0.7941 (3)	0.0228 (2)	0.3204 (2)	0.0488 (7)	
H45	0.8121	−0.0407	0.3004	0.059*	
C46	0.6794 (2)	0.04100 (19)	0.3205 (2)	0.0441 (6)	
H46	0.6206	−0.0094	0.3	0.053*	
C47	0.4769 (2)	0.23407 (19)	0.3576 (2)	0.0428 (6)	
C48	0.3514 (3)	0.2177 (2)	0.3524 (3)	0.0518 (7)	
H48	0.3262	0.1496	0.3481	0.062*	
C49	0.2708 (3)	0.2873 (2)	0.3530 (3)	0.0578 (8)	
H49	0.1977	0.2596	0.3505	0.069*	
C50	0.2750 (3)	0.4020 (2)	0.3570 (2)	0.0538 (7)	
C51	0.9488 (3)	0.2750 (2)	0.4126 (3)	0.0619 (9)	
H51A	0.9188	0.3299	0.4405	0.093*	
H51B	0.9664	0.3009	0.3599	0.093*	
H51C	1.0204	0.2475	0.4576	0.093*	
C52	1.0043 (3)	0.0740 (3)	0.3436 (3)	0.0600 (8)	
H52A	1.0097	0.0021	0.3313	0.09*	
H52B	1.0652	0.0917	0.4017	0.09*	
H52C	1.0154	0.1137	0.2941	0.09*	
N3	0.53418 (19)	0.14672 (16)	0.35181 (16)	0.0420 (5)	
H3N	0.4942	0.0907	0.3476	0.05*	
O41	0.52359 (19)	0.31979 (15)	0.3658 (2)	0.0668 (7)	
O42	0.3700 (2)	0.45152 (16)	0.3612 (2)	0.0663 (7)	
H42A	0.4274	0.4076	0.3639	0.08*	
O43	0.1861 (2)	0.44801 (18)	0.3567 (2)	0.0835 (9)	
C61	−0.1341 (3)	0.8982 (2)	0.11567 (19)	0.0422 (6)	
C62	−0.2221 (3)	0.9730 (2)	0.0861 (2)	0.0452 (6)	
H62	−0.2043	1.0366	0.0661	0.054*	
C63	−0.3375 (3)	0.9533 (2)	0.0862 (2)	0.0468 (6)	
C64	−0.3650 (3)	0.8579 (2)	0.1163 (2)	0.0489 (7)	
C65	−0.2730 (3)	0.7836 (2)	0.1469 (2)	0.0507 (7)	
H65	−0.2894	0.7198	0.1673	0.061*	
C66	−0.1596 (3)	0.8041 (2)	0.1469 (2)	0.0502 (7)	
H66	−0.0998	0.7546	0.1679	0.06*	
C67	0.0405 (3)	0.9968 (2)	0.1050 (2)	0.0466 (7)	
C68	0.1659 (3)	0.9812 (2)	0.1108 (2)	0.0523 (8)	
H68	0.1915	0.9132	0.115	0.063*	
C69	0.2463 (3)	1.0512 (2)	0.1108 (3)	0.0615 (8)	
H69	0.3196	1.0235	0.1137	0.074*	
C70	0.2430 (3)	1.1660 (2)	0.1070 (3)	0.0554 (8)	
C71	−0.4309 (3)	1.0354 (3)	0.0559 (3)	0.0679 (9)	
H71A	−0.4987	1.0102	0.0055	0.102*	
H71B	−0.3985	1.0946	0.0354	0.102*	
H71C	−0.4555	1.0542	0.1074	0.102*	
C72	−0.4875 (3)	0.8336 (3)	0.1182 (3)	0.0653 (9)	
H72A	−0.4884	0.7648	0.1407	0.098*	0.5
H72B	−0.5452	0.8394	0.0562	0.098*	0.5
H72C	−0.5074	0.8812	0.1587	0.098*	0.5
H72D	−0.5389	0.8921	0.0964	0.098*	0.5
H72E	−0.4821	0.8175	0.1809	0.098*	0.5
H72F	−0.5199	0.7757	0.0784	0.098*	0.5
N4	−0.0147 (2)	0.91139 (17)	0.11665 (17)	0.0450 (5)	
H4N	0.0278	0.8563	0.1261	0.054*	
O61	−0.0095 (2)	1.08224 (16)	0.0903 (2)	0.0649 (6)	
O62	0.1442 (2)	1.21427 (16)	0.09843 (19)	0.0637 (6)	
H62A	0.0882	1.1694	0.0969	0.076*	
O63	0.3342 (2)	1.21084 (19)	0.1144 (3)	0.0878 (9)	

### Atomic displacement parameters (Å^2^)

**Table:**

	*U*^11^	*U*^22^	*U*^33^	*U*^12^	*U*^13^	*U*^23^
C1	0.0381 (14)	0.0391 (13)	0.0533 (15)	−0.0002 (11)	0.0177 (12)	−0.0032 (11)
C2	0.0439 (16)	0.0392 (14)	0.0527 (16)	−0.0027 (12)	0.0178 (13)	0.0025 (12)
C3	0.0528 (18)	0.0398 (14)	0.0507 (17)	−0.0023 (12)	0.0238 (14)	−0.0044 (11)
C4	0.0418 (16)	0.0443 (15)	0.0589 (17)	0.0038 (12)	0.0209 (14)	−0.0069 (12)
C5	0.0468 (16)	0.0394 (14)	0.0616 (18)	0.0040 (12)	0.0187 (15)	0.0006 (13)
C6	0.0461 (17)	0.0347 (13)	0.0676 (19)	0.0014 (12)	0.0240 (14)	0.0011 (12)
C7	0.0356 (14)	0.0372 (14)	0.0689 (18)	0.0015 (11)	0.0196 (13)	0.0007 (12)
C8	0.0403 (16)	0.0445 (16)	0.085 (2)	−0.0039 (13)	0.0220 (16)	−0.0029 (15)
C9	0.0415 (16)	0.0452 (16)	0.093 (2)	−0.0009 (13)	0.0282 (16)	0.0067 (15)
C10	0.0465 (16)	0.0411 (15)	0.0701 (19)	0.0039 (13)	0.0209 (14)	0.0039 (13)
C11	0.0536 (18)	0.0518 (17)	0.082 (2)	−0.0080 (14)	0.0370 (17)	−0.0011 (15)
C12	0.0453 (18)	0.065 (2)	0.080 (2)	0.0038 (15)	0.0249 (17)	−0.0128 (16)
N1	0.0429 (13)	0.0365 (12)	0.0731 (16)	−0.0020 (10)	0.0253 (12)	−0.0008 (11)
O1	0.0457 (11)	0.0383 (10)	0.1082 (18)	−0.0021 (9)	0.0386 (12)	−0.0003 (11)
O2	0.0640 (16)	0.0378 (12)	0.137 (2)	0.0040 (10)	0.0545 (16)	0.0031 (12)
O3	0.0603 (14)	0.0574 (14)	0.1057 (18)	0.0152 (11)	0.0428 (14)	0.0056 (12)
C21	0.0358 (13)	0.0307 (12)	0.0480 (15)	0.0021 (10)	0.0194 (11)	−0.0004 (10)
C22	0.0402 (14)	0.0299 (12)	0.0512 (15)	−0.0014 (10)	0.0220 (12)	−0.0030 (10)
C23	0.0389 (14)	0.0327 (12)	0.0469 (15)	0.0058 (10)	0.0197 (12)	0.0027 (10)
C24	0.0396 (14)	0.0398 (14)	0.0453 (14)	−0.0043 (11)	0.0187 (12)	0.0004 (11)
C25	0.0519 (18)	0.0355 (13)	0.0623 (19)	−0.0108 (12)	0.0270 (15)	−0.0101 (12)
C26	0.0515 (17)	0.0333 (13)	0.0658 (18)	−0.0012 (11)	0.0323 (14)	−0.0067 (11)
C27	0.0430 (15)	0.0326 (13)	0.0592 (17)	0.0004 (11)	0.0256 (13)	−0.0017 (11)
C28	0.0410 (15)	0.0294 (12)	0.087 (2)	0.0030 (11)	0.0293 (15)	−0.0044 (13)
C29	0.0320 (14)	0.0447 (16)	0.095 (2)	−0.0003 (12)	0.0291 (15)	−0.0055 (15)
C30	0.0359 (15)	0.0408 (15)	0.078 (2)	−0.0089 (12)	0.0219 (14)	−0.0089 (13)
C31	0.0512 (17)	0.0390 (14)	0.076 (2)	0.0089 (12)	0.0335 (16)	−0.0027 (13)
C32	0.0438 (16)	0.0537 (17)	0.0700 (19)	−0.0067 (14)	0.0238 (15)	−0.0015 (14)
N2	0.0338 (11)	0.0292 (10)	0.0679 (15)	0.0024 (8)	0.0249 (11)	−0.0017 (9)
O21	0.0489 (11)	0.0289 (9)	0.1141 (18)	0.0038 (8)	0.0455 (12)	−0.0007 (10)
O22	0.0547 (15)	0.0347 (11)	0.137 (2)	−0.0010 (9)	0.0542 (16)	0.0025 (12)
O23	0.0545 (13)	0.0493 (12)	0.1149 (19)	−0.0133 (11)	0.0444 (13)	−0.0027 (12)
C41	0.0361 (14)	0.0295 (11)	0.0482 (15)	0.0044 (10)	0.0190 (11)	0.0018 (10)
C42	0.0459 (15)	0.0303 (12)	0.0509 (15)	0.0042 (10)	0.0207 (12)	−0.0038 (10)
C43	0.0354 (13)	0.0326 (12)	0.0511 (15)	0.0018 (10)	0.0140 (12)	−0.0004 (11)
C44	0.0352 (14)	0.0378 (13)	0.0527 (15)	0.0093 (10)	0.0191 (12)	0.0015 (11)
C45	0.0478 (16)	0.0313 (12)	0.072 (2)	0.0049 (11)	0.0273 (15)	−0.0074 (12)
C46	0.0387 (14)	0.0286 (12)	0.0659 (18)	0.0019 (10)	0.0191 (13)	−0.0042 (11)
C47	0.0380 (14)	0.0272 (12)	0.0672 (18)	0.0044 (10)	0.0231 (13)	0.0005 (11)
C48	0.0461 (16)	0.0309 (13)	0.088 (2)	−0.0034 (12)	0.0348 (16)	−0.0037 (13)
C49	0.0366 (15)	0.0439 (16)	0.102 (2)	−0.0010 (13)	0.0352 (16)	−0.0026 (16)
C50	0.0421 (16)	0.0385 (15)	0.086 (2)	0.0077 (12)	0.0284 (15)	−0.0023 (13)
C51	0.0411 (16)	0.0489 (17)	0.094 (3)	−0.0070 (12)	0.0208 (17)	−0.0182 (16)
C52	0.0401 (17)	0.0526 (17)	0.095 (2)	0.0091 (13)	0.0330 (16)	−0.0054 (16)
N3	0.0379 (12)	0.0271 (10)	0.0672 (15)	0.0036 (9)	0.0263 (11)	−0.0011 (9)
O41	0.0426 (11)	0.0301 (10)	0.138 (2)	0.0009 (9)	0.0438 (13)	−0.0023 (11)
O42	0.0481 (13)	0.0353 (11)	0.124 (2)	0.0050 (9)	0.0399 (14)	−0.0040 (11)
O43	0.0504 (14)	0.0443 (12)	0.166 (3)	0.0117 (10)	0.0500 (17)	−0.0096 (14)
C61	0.0403 (15)	0.0354 (13)	0.0528 (16)	−0.0049 (11)	0.0187 (13)	−0.0022 (11)
C62	0.0409 (14)	0.0365 (13)	0.0579 (17)	−0.0006 (11)	0.0167 (13)	0.0060 (12)
C63	0.0440 (15)	0.0406 (14)	0.0541 (16)	0.0000 (12)	0.0148 (13)	0.0023 (12)
C64	0.0454 (15)	0.0376 (14)	0.0648 (18)	−0.0070 (11)	0.0206 (14)	−0.0049 (12)
C65	0.0520 (16)	0.0375 (14)	0.067 (2)	−0.0064 (12)	0.0262 (16)	0.0011 (13)
C66	0.0562 (17)	0.0351 (14)	0.0647 (18)	0.0001 (12)	0.0279 (15)	0.0029 (12)
C67	0.0427 (16)	0.0385 (14)	0.0654 (18)	−0.0033 (11)	0.0273 (14)	−0.0005 (12)
C68	0.0423 (16)	0.0370 (14)	0.085 (2)	0.0015 (12)	0.0310 (15)	0.0033 (14)
C69	0.0459 (17)	0.0450 (17)	0.101 (3)	−0.0004 (14)	0.0351 (17)	0.0058 (17)
C70	0.0466 (17)	0.0437 (16)	0.078 (2)	−0.0024 (14)	0.0241 (15)	0.0061 (14)
C71	0.0435 (17)	0.0501 (17)	0.108 (3)	0.0048 (13)	0.0232 (18)	0.0119 (17)
C72	0.0508 (18)	0.0541 (19)	0.091 (3)	−0.0094 (15)	0.0245 (18)	−0.0014 (17)
N4	0.0408 (12)	0.0311 (11)	0.0658 (15)	0.0031 (9)	0.0220 (12)	0.0025 (9)
O61	0.0446 (11)	0.0361 (10)	0.1192 (19)	0.0011 (9)	0.0348 (12)	0.0126 (11)
O62	0.0484 (13)	0.0386 (11)	0.1059 (18)	0.0005 (9)	0.0288 (13)	0.0048 (11)
O63	0.0550 (15)	0.0537 (14)	0.164 (3)	−0.0110 (11)	0.0502 (17)	0.0081 (16)

### Geometric parameters (Å, °)

**Table:**

C1—C6	1.378 (4)	C41—C42	1.384 (4)
C1—C2	1.390 (4)	C41—C46	1.397 (4)
C1—N1	1.423 (4)	C41—N3	1.420 (3)
C2—C3	1.399 (5)	C42—C43	1.374 (4)
C2—H2	0.93	C42—H42	0.93
C3—C4	1.385 (4)	C43—C44	1.389 (4)
C3—C11	1.505 (4)	C43—C51	1.516 (4)
C4—C5	1.395 (4)	C44—C45	1.379 (4)
C4—C12	1.498 (4)	C44—C52	1.518 (4)
C5—C6	1.388 (4)	C45—C46	1.386 (4)
C5—H5	0.93	C45—H45	0.93
C6—H6	0.93	C46—H46	0.93
C7—O1	1.237 (3)	C47—O41	1.232 (3)
C7—N1	1.341 (4)	C47—N3	1.343 (3)
C7—C8	1.483 (4)	C47—C48	1.483 (4)
C8—C9	1.322 (5)	C48—C49	1.320 (4)
C8—H8	0.93	C48—H48	0.93
C9—C10	1.505 (4)	C49—C50	1.493 (4)
C9—H9	0.93	C49—H49	0.93
C10—O3	1.206 (4)	C50—O43	1.214 (3)
C10—O2	1.306 (4)	C50—O42	1.283 (4)
C11—H11A	0.96	C51—H51A	0.96
C11—H11B	0.96	C51—H51B	0.96
C11—H11C	0.96	C51—H51C	0.96
C12—H12A	0.96	C52—H52A	0.96
C12—H12B	0.96	C52—H52B	0.96
C12—H12C	0.96	C52—H52C	0.96
C12—H12D	0.96	N3—H3N	0.86
C12—H12E	0.96	O42—H42A	0.88
C12—H12F	0.96	C61—C66	1.384 (4)
N1—H1	0.86	C61—C62	1.385 (4)
O2—H2A	0.88	C61—N4	1.426 (4)
C21—C26	1.384 (4)	C62—C63	1.398 (4)
C21—C22	1.395 (3)	C62—H62	0.93
C21—N2	1.420 (3)	C63—C64	1.400 (4)
C22—C23	1.394 (4)	C63—C71	1.494 (4)
C22—H22	0.93	C64—C65	1.413 (4)
C23—C24	1.406 (4)	C64—C72	1.502 (4)
C23—C31	1.500 (4)	C65—C66	1.377 (5)
C24—C25	1.390 (4)	C65—H65	0.93
C24—C32	1.505 (4)	C66—H66	0.93
C25—C26	1.375 (4)	C67—O61	1.243 (3)
C25—H25	0.93	C67—N4	1.333 (3)
C26—H26	0.93	C67—C68	1.479 (4)
C27—O21	1.247 (3)	C68—C69	1.321 (4)
C27—N2	1.332 (3)	C68—H68	0.93
C27—C28	1.483 (4)	C69—C70	1.493 (4)
C28—C29	1.343 (4)	C69—H69	0.93
C28—H28	0.93	C70—O63	1.203 (4)
C29—C30	1.467 (4)	C70—O62	1.299 (4)
C29—H29	0.93	C71—H71A	0.96
C30—O23	1.218 (4)	C71—H71B	0.96
C30—O22	1.301 (4)	C71—H71C	0.96
C31—H31A	0.96	C72—H72A	0.96
C31—H31B	0.96	C72—H72B	0.96
C31—H31C	0.96	C72—H72C	0.96
C32—H32A	0.96	C72—H72D	0.96
C32—H32B	0.96	C72—H72E	0.96
C32—H32C	0.96	C72—H72F	0.96
N2—H2N	0.86	N4—H4N	0.86
O22—H22A	0.88	O62—H62A	0.88
			
C6—C1—C2	119.2 (3)	C42—C41—C46	119.2 (2)
C6—C1—N1	116.0 (3)	C42—C41—N3	125.4 (2)
C2—C1—N1	124.8 (3)	C46—C41—N3	115.4 (2)
C1—C2—C3	120.2 (3)	C43—C42—C41	121.0 (2)
C1—C2—H2	119.9	C43—C42—H42	119.5
C3—C2—H2	119.9	C41—C42—H42	119.5
C4—C3—C2	120.5 (3)	C42—C43—C44	120.2 (2)
C4—C3—C11	121.0 (3)	C42—C43—C51	119.0 (2)
C2—C3—C11	118.5 (3)	C44—C43—C51	120.9 (2)
C3—C4—C5	118.9 (3)	C45—C44—C43	119.1 (2)
C3—C4—C12	121.6 (3)	C45—C44—C52	118.7 (2)
C5—C4—C12	119.5 (3)	C43—C44—C52	122.2 (3)
C6—C5—C4	120.4 (3)	C44—C45—C46	121.3 (2)
C6—C5—H5	119.8	C44—C45—H45	119.4
C4—C5—H5	119.8	C46—C45—H45	119.4
C1—C6—C5	120.8 (3)	C45—C46—C41	119.3 (2)
C1—C6—H6	119.6	C45—C46—H46	120.3
C5—C6—H6	119.6	C41—C46—H46	120.3
O1—C7—N1	123.5 (3)	O41—C47—N3	123.3 (3)
O1—C7—C8	122.9 (3)	O41—C47—C48	123.1 (2)
N1—C7—C8	113.6 (2)	N3—C47—C48	113.6 (2)
C9—C8—C7	128.5 (3)	C49—C48—C47	128.5 (3)
C9—C8—H8	115.8	C49—C48—H48	115.8
C7—C8—H8	115.8	C47—C48—H48	115.8
C8—C9—C10	131.9 (3)	C48—C49—C50	132.1 (3)
C8—C9—H9	114	C48—C49—H49	114
C10—C9—H9	114	C50—C49—H49	114
O3—C10—O2	121.1 (3)	O43—C50—O42	120.4 (3)
O3—C10—C9	119.2 (3)	O43—C50—C49	118.3 (3)
O2—C10—C9	119.6 (3)	O42—C50—C49	121.3 (3)
C3—C11—H11A	109.5	C43—C51—H51A	109.5
C3—C11—H11B	109.5	C43—C51—H51B	109.5
H11A—C11—H11B	109.5	H51A—C51—H51B	109.5
C3—C11—H11C	109.5	C43—C51—H51C	109.5
H11A—C11—H11C	109.5	H51A—C51—H51C	109.5
H11B—C11—H11C	109.5	H51B—C51—H51C	109.5
C4—C12—H12A	109.5	C44—C52—H52A	109.5
C4—C12—H12B	109.5	C44—C52—H52B	109.5
H12A—C12—H12B	109.5	H52A—C52—H52B	109.5
C4—C12—H12C	109.5	C44—C52—H52C	109.5
H12A—C12—H12C	109.5	H52A—C52—H52C	109.5
H12B—C12—H12C	109.5	H52B—C52—H52C	109.5
C4—C12—H12D	109.5	C47—N3—C41	127.8 (2)
H12A—C12—H12D	141.1	C47—N3—H3N	116.1
H12B—C12—H12D	56.3	C41—N3—H3N	116.1
H12C—C12—H12D	56.3	C50—O42—H42A	109.5
C4—C12—H12E	109.5	C66—C61—C62	120.1 (3)
H12A—C12—H12E	56.3	C66—C61—N4	116.0 (3)
H12B—C12—H12E	141.1	C62—C61—N4	123.9 (2)
H12C—C12—H12E	56.3	C61—C62—C63	120.2 (3)
H12D—C12—H12E	109.5	C61—C62—H62	119.9
C4—C12—H12F	109.5	C63—C62—H62	119.9
H12A—C12—H12F	56.3	C62—C63—C64	120.2 (3)
H12B—C12—H12F	56.3	C62—C63—C71	119.7 (3)
H12C—C12—H12F	141.1	C64—C63—C71	120.1 (3)
H12D—C12—H12F	109.5	C63—C64—C65	118.2 (3)
H12E—C12—H12F	109.5	C63—C64—C72	122.0 (3)
C7—N1—C1	128.4 (2)	C65—C64—C72	119.8 (3)
C7—N1—H1	115.8	C66—C65—C64	121.0 (3)
C1—N1—H1	115.8	C66—C65—H65	119.5
C10—O2—H2A	109.5	C64—C65—H65	119.5
C26—C21—C22	119.6 (2)	C65—C66—C61	120.2 (3)
C26—C21—N2	116.7 (2)	C65—C66—H66	119.9
C22—C21—N2	123.7 (2)	C61—C66—H66	119.9
C23—C22—C21	120.3 (2)	O61—C67—N4	122.8 (3)
C23—C22—H22	119.9	O61—C67—C68	123.0 (2)
C21—C22—H22	119.9	N4—C67—C68	114.3 (2)
C22—C23—C24	120.0 (2)	C69—C68—C67	128.4 (3)
C22—C23—C31	119.8 (2)	C69—C68—H68	115.8
C24—C23—C31	120.2 (2)	C67—C68—H68	115.8
C25—C24—C23	118.2 (2)	C68—C69—C70	132.8 (3)
C25—C24—C32	120.2 (3)	C68—C69—H69	113.6
C23—C24—C32	121.5 (2)	C70—C69—H69	113.6
C26—C25—C24	121.8 (3)	O63—C70—O62	122.1 (3)
C26—C25—H25	119.1	O63—C70—C69	118.0 (3)
C24—C25—H25	119.1	O62—C70—C69	119.9 (3)
C25—C26—C21	120.0 (2)	C63—C71—H71A	109.5
C25—C26—H26	120	C63—C71—H71B	109.5
C21—C26—H26	120	H71A—C71—H71B	109.5
O21—C27—N2	123.2 (3)	C63—C71—H71C	109.5
O21—C27—C28	122.7 (2)	H71A—C71—H71C	109.5
N2—C27—C28	114.1 (2)	H71B—C71—H71C	109.5
C29—C28—C27	127.6 (3)	C64—C72—H72A	109.5
C29—C28—H28	116.2	C64—C72—H72B	109.5
C27—C28—H28	116.2	H72A—C72—H72B	109.5
C28—C29—C30	132.9 (3)	C64—C72—H72C	109.5
C28—C29—H29	113.6	H72A—C72—H72C	109.5
C30—C29—H29	113.6	H72B—C72—H72C	109.5
O23—C30—O22	120.5 (3)	C64—C72—H72D	109.5
O23—C30—C29	119.1 (3)	H72A—C72—H72D	141.1
O22—C30—C29	120.3 (3)	H72B—C72—H72D	56.3
C23—C31—H31A	109.5	H72C—C72—H72D	56.3
C23—C31—H31B	109.5	C64—C72—H72E	109.5
H31A—C31—H31B	109.5	H72A—C72—H72E	56.3
C23—C31—H31C	109.5	H72B—C72—H72E	141.1
H31A—C31—H31C	109.5	H72C—C72—H72E	56.3
H31B—C31—H31C	109.5	H72D—C72—H72E	109.5
C24—C32—H32A	109.5	C64—C72—H72F	109.5
C24—C32—H32B	109.5	H72A—C72—H72F	56.3
H32A—C32—H32B	109.5	H72B—C72—H72F	56.3
C24—C32—H32C	109.5	H72C—C72—H72F	141.1
H32A—C32—H32C	109.5	H72D—C72—H72F	109.5
H32B—C32—H32C	109.5	H72E—C72—H72F	109.5
C27—N2—C21	128.7 (2)	C67—N4—C61	129.3 (2)
C27—N2—H2N	115.7	C67—N4—H4N	115.4
C21—N2—H2N	115.7	C61—N4—H4N	115.4
C30—O22—H22A	109.5	C70—O62—H62A	109.5
			
C6—C1—C2—C3	−1.3 (4)	C46—C41—C42—C43	2.2 (4)
N1—C1—C2—C3	179.2 (3)	N3—C41—C42—C43	179.5 (3)
C1—C2—C3—C4	0.9 (4)	C41—C42—C43—C44	−1.8 (4)
C1—C2—C3—C11	−179.8 (3)	C41—C42—C43—C51	177.8 (3)
C2—C3—C4—C5	−0.6 (4)	C42—C43—C44—C45	0.0 (4)
C11—C3—C4—C5	−179.9 (3)	C51—C43—C44—C45	−179.6 (3)
C2—C3—C4—C12	179.8 (3)	C42—C43—C44—C52	178.8 (3)
C11—C3—C4—C12	0.4 (4)	C51—C43—C44—C52	−0.8 (4)
C3—C4—C5—C6	0.6 (5)	C43—C44—C45—C46	1.4 (5)
C12—C4—C5—C6	−179.7 (3)	C52—C44—C45—C46	−177.4 (3)
C2—C1—C6—C5	1.4 (4)	C44—C45—C46—C41	−1.0 (5)
N1—C1—C6—C5	−179.1 (3)	C42—C41—C46—C45	−0.8 (4)
C4—C5—C6—C1	−1.1 (5)	N3—C41—C46—C45	−178.4 (3)
O1—C7—C8—C9	−13.3 (6)	O41—C47—C48—C49	1.8 (6)
N1—C7—C8—C9	167.3 (4)	N3—C47—C48—C49	−177.6 (4)
C7—C8—C9—C10	2.3 (8)	C47—C48—C49—C50	1.4 (7)
C8—C9—C10—O3	−171.1 (4)	C48—C49—C50—O43	−179.9 (4)
C8—C9—C10—O2	10.9 (7)	C48—C49—C50—O42	−0.4 (7)
O1—C7—N1—C1	6.8 (5)	O41—C47—N3—C41	−1.5 (5)
C8—C7—N1—C1	−173.9 (3)	C48—C47—N3—C41	178.0 (3)
C6—C1—N1—C7	159.6 (3)	C42—C41—N3—C47	18.9 (4)
C2—C1—N1—C7	−20.9 (5)	C46—C41—N3—C47	−163.7 (3)
C26—C21—C22—C23	−0.1 (4)	C66—C61—C62—C63	−1.0 (5)
N2—C21—C22—C23	−179.9 (2)	N4—C61—C62—C63	179.1 (3)
C21—C22—C23—C24	−0.8 (4)	C61—C62—C63—C64	0.1 (5)
C21—C22—C23—C31	180.0 (3)	C61—C62—C63—C71	178.8 (3)
C22—C23—C24—C25	1.4 (4)	C62—C63—C64—C65	0.5 (5)
C31—C23—C24—C25	−179.3 (3)	C71—C63—C64—C65	−178.2 (3)
C22—C23—C24—C32	−178.0 (3)	C62—C63—C64—C72	179.6 (3)
C31—C23—C24—C32	1.3 (4)	C71—C63—C64—C72	1.0 (5)
C23—C24—C25—C26	−1.2 (5)	C63—C64—C65—C66	−0.2 (5)
C32—C24—C25—C26	178.2 (3)	C72—C64—C65—C66	−179.3 (3)
C24—C25—C26—C21	0.3 (5)	C64—C65—C66—C61	−0.7 (5)
C22—C21—C26—C25	0.4 (4)	C62—C61—C66—C65	1.3 (5)
N2—C21—C26—C25	−179.9 (3)	N4—C61—C66—C65	−178.8 (3)
O21—C27—C28—C29	−9.7 (6)	O61—C67—C68—C69	7.0 (6)
N2—C27—C28—C29	170.8 (4)	N4—C67—C68—C69	−173.2 (4)
C27—C28—C29—C30	−0.3 (7)	C67—C68—C69—C70	1.3 (8)
C28—C29—C30—O23	−170.8 (4)	C68—C69—C70—O63	175.1 (5)
C28—C29—C30—O22	9.5 (7)	C68—C69—C70—O62	−3.2 (7)
O21—C27—N2—C21	7.0 (5)	O61—C67—N4—C61	−0.3 (5)
C28—C27—N2—C21	−173.6 (3)	C68—C67—N4—C61	179.8 (3)
C26—C21—N2—C27	155.7 (3)	C66—C61—N4—C67	−168.4 (3)
C22—C21—N2—C27	−24.5 (4)	C62—C61—N4—C67	11.4 (5)

### Hydrogen-bond geometry (Å, °)

**Table:**

*D*—H···*A*	*D*—H	H···*A*	*D*···*A*	*D*—H···*A*
N1—H1···O63^i^	0.86	2.00	2.851 (3)	168
N2—H2N···O43	0.86	2.03	2.865 (3)	163
N3—H3N···O23^i^	0.86	2.07	2.916 (3)	167
N4—H4N···O3	0.86	2.08	2.930 (3)	169
O2—H2A···O1	0.88	1.62	2.481 (3)	165
O22—H22A···O21	0.88	1.59	2.471 (3)	176
O42—H42A···O41	0.88	1.61	2.487 (3)	175
O62—H62A···O61	0.88	1.6	2.480 (3)	176

Symmetry codes: (i) *x*, *y*−1, *z*.
